# 1-(1*H*-1,2,3-Benzotriazol-1-yl)-2-(4-meth­oxy­phen­yl)ethanone

**DOI:** 10.1107/S1600536812043759

**Published:** 2012-10-27

**Authors:** Abdullah M. Asiri, Nader E. Abo-Dya, Muhammad Nadeem Arshad, Khalid A. Alamry, Muhammad Shafiq

**Affiliations:** a Chemistry Department, Faculty of Science, King Abdulaziz University PO Box 80203 , Jeddah 21589, Saudi Arabia; b Center of Excellence for Advanced Materials Research (CEAMR), Faculty of Science, King Abdulaziz University PO Box 80203, Jeddah 21589, Saudi Arabia; c Department of Pharmaceutical Organic Chemistry, Faculty of Pharmacy, Zagazig University, Zagazig, 44519, Egypt; d Department of Chemistry, Government College University, Faisalabad 38040, Pakistan

## Abstract

In the title compound, C_15_H_13_N_3_O_2_, the dihedral angle between the benzotriazole ring system (r.m.s. deviation = 0.0124 Å) and the benzene ring is 76.21 (3)°. The meth­oxy C atom deviates from its benzene ring plane by 0.063 (2)Å. In the crystal, inversion dimers linked by pairs of C—H⋯O hydrogen bonds generate *R*_2_^2^(12) loops.

## Related literature

For chemical background, see: Katritzky *et al.* (1996*a* ▶,*b* ▶, 2005 ▶, 2010 ▶). For a related structure, see: Selvarathy Grace *et al.* (2012 ▶). For related literature, see: Zou *et al.* (2006 ▶).
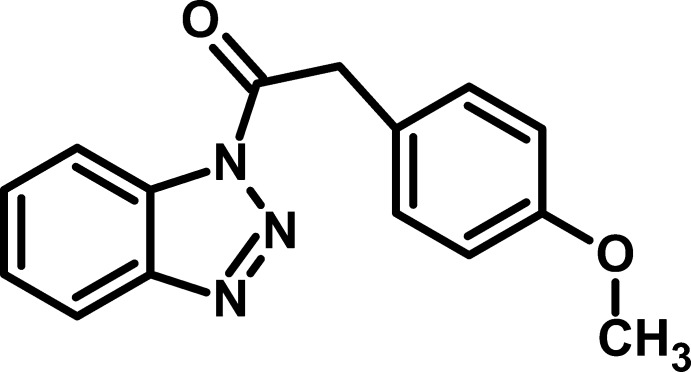


## Experimental

### 

#### Crystal data

C_15_H_13_N_3_O_2_
*M* *_r_* = 267.28
Monoclinic, 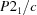

*a* = 5.4209 (1) Å
*b* = 24.4894 (5) Å
*c* = 10.0555 (2) Å
β = 98.552 (2)°
*V* = 1320.07 (4) Å^3^
*Z* = 4
Cu *K*α radiation
μ = 0.75 mm^−1^
*T* = 296 K
0.34 × 0.17 × 0.16 mm

#### Data collection

Agilent SuperNova (Dual, Cu at zero, Atlas CCD) diffractometer
Absorption correction: multi-scan (*CrysAlis PRO*; Agilent, 2012 ▶) *T*_min_ = 0.784, *T*_max_ = 0.889
6122 measured reflections
2707 independent reflections
2340 reflections with *I* > 2σ(*I*)
*R*_int_ = 0.019

#### Refinement

*R*[*F* ^2^ > 2σ(*F* ^2^)] = 0.039
*wR*(*F* ^2^) = 0.109
*S* = 1.08
2707 reflections
182 parameters
H-atom parameters constrained
Δρ_max_ = 0.14 e Å^−3^
Δρ_min_ = −0.16 e Å^−3^

### 

Data collection: *CrysAlis PRO* (Agilent, 2012 ▶); cell refinement: *CrysAlis PRO*; data reduction: *CrysAlis PRO*; program(s) used to solve structure: *SHELXS97* (Sheldrick, 2008 ▶); program(s) used to refine structure: *SHELXL97* (Sheldrick, 2008 ▶); molecular graphics: *PLATON* (Spek, 2009 ▶); software used to prepare material for publication: *WinGX* (Farrugia, 1999 ▶) and *X-SEED* (Barbour, 2001 ▶).

## Supplementary Material

Crystal structure: contains datablock(s) I, global. DOI: 10.1107/S1600536812043759/hb6975sup1.cif

Structure factors: contains datablock(s) I. DOI: 10.1107/S1600536812043759/hb6975Isup2.hkl

Supplementary material file. DOI: 10.1107/S1600536812043759/hb6975Isup3.cml

Additional supplementary materials:  crystallographic information; 3D view; checkCIF report

## Figures and Tables

**Table 1:** Hydrogen-bond geometry (Å, °)

*D*—H⋯*A*	*D*—H	H⋯*A*	*D*⋯*A*	*D*—H⋯*A*
C5—H5⋯O1^i^	0.93	2.40	3.1912 (16)	143

Symmetry code: (i) 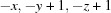.

**Fig. 1.**
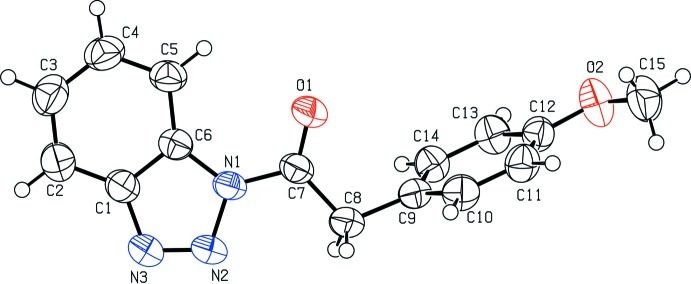
The molecular structure of (I) with 50% displacement ellipsoids.

**Fig. 2.**
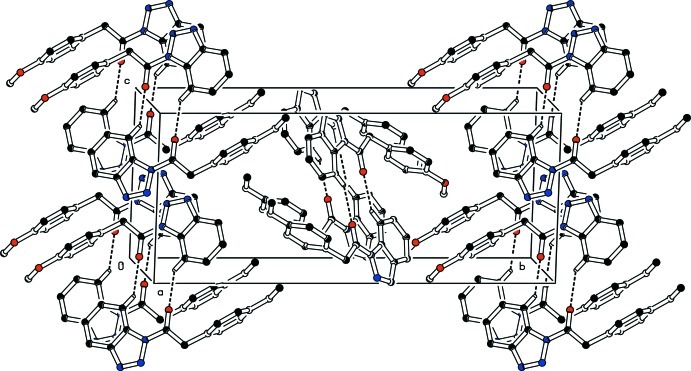
Unit cell packing diagram showing intermolecular hydrogen bonds, drawn using dashed lines. Hydrogen atoms not involved in bonding have been omitted for clarity.

## supplementary crystallographic information

### Comment

*N*-Acylbenzotriazoles are mild, regioselective and regiospecific reagents for *N*-, *O*-, *C*-, and *S*-acylation (Katritzky *et al.*, 2010), & (Katritzky *et al.*, 1996*a*). The title compound was previously converted into of a 1,3-diarylacetone (Katritzky *et al.*, 2005) and an aryl benzyl sulfoxide (Katritzky *et al.*, 1996*b*).

The title coompound is related in structure with 1-benzyl-1*H*-benzotriazole (Selvarathy Grace *et al.*, 2012). The benzotriazole ring is almost planer with r.m.s. deviation of fitted non-hydrogen atoms (C1—C6/N1/N2/N3) is 0.0124 Å. The oxygen atom of carbonyl group is displaced at 0.0724 (2) Å with respect to benzotriazole. The methoxy benzene ring (C9—C14) is orientedted at dihedral angle of 76.21 (3)° with respect to benzotriazole rings. The C—H···O type weak hydrogen bonding interaction results in dimers about inversion center and generate twelve membered ring motif *R*_2_^2^(12) (Table. 1, Fig. 2).

### Experimental

A solution of thionyl chloride (0.4 ml, 5.5 mmol) and benzotriazole (1.79 g., 15 mmol) in methylene chloride (30 ml) was stirred at 293 K for 30 minutes. 2-(4-methoxypheny)acetic acid (0.83 g., 5 mmol) was then added and the heterogeneous mixture was stirred for 2 hr. The solid was filtered and methylene chloride (50mL) was added to the filtrate. The organic layer was extracted with saturated Na_2_CO_3_ (3 × 15 ml), brine (2 × 5 ml) and dried over anhyd. Na_2_SO_4_. Evaporation of methylene chloride solution afforded colourless prisms (1.21 g., 90% yield).

### Refinement

All the C—H and H-atoms were positioned with idealized geometry with C—H = 0.93 Å for aromatic, C—H = 0.97 Å for methylene & C—H = 0.96 Å for methyl groups. H-atoms were refined as riding with *U*_iso_(H) = k*U*_eq_(C, N), where k = 1.2 for aromatic & methylene and k = 1.5 for methyl H-atoms.

### Crystal data

**Table:**

C_15_H_13_N_3_O_2_	*F*(000) = 560
*M**_r_* = 267.28	*D*_x_ = 1.345 Mg m^−^^3^
Monoclinic, *P*2_1_/*c*	Cu *K*α radiation, λ = 1.54184 Å
Hall symbol: -P 2ybc	Cell parameters from 3661 reflections
*a* = 5.4209 (1) Å	θ = 4.4–76.0°
*b* = 24.4894 (5) Å	µ = 0.75 mm^−^^1^
*c* = 10.0555 (2) Å	*T* = 296 K
β = 98.552 (2)°	Prismatic, colorless
*V* = 1320.07 (4) Å^3^	0.34 × 0.17 × 0.16 mm
*Z* = 4	

### Data collection

**Table:**

Agilent SuperNova (Dual, Cu at zero, Atlas CCD) diffractometer	2707 independent reflections
Radiation source: SuperNova (Cu) X-ray Source	2340 reflections with *I* > 2σ(*I*)
Mirror monochromator	*R*_int_ = 0.019
ω scans	θ_max_ = 76.2°, θ_min_ = 4.8°
Absorption correction: multi-scan (*CrysAlis PRO*; Agilent, 2012)	*h* = −6→4
*T*_min_ = 0.784, *T*_max_ = 0.889	*k* = −29→30
6122 measured reflections	*l* = −12→12

### Refinement

**Table:**

Refinement on *F*^2^	Primary atom site location: structure-invariant direct methods
Least-squares matrix: full	Secondary atom site location: difference Fourier map
*R*[*F*^2^ > 2σ(*F*^2^)] = 0.039	Hydrogen site location: inferred from neighbouring sites
*wR*(*F*^2^) = 0.109	H-atom parameters constrained
*S* = 1.08	*w* = 1/[σ^2^(*F*_o_^2^) + (0.0527*P*)^2^ + 0.1534*P*] where *P* = (*F*_o_^2^ + 2*F*_c_^2^)/3
2707 reflections	(Δ/σ)_max_ < 0.001
182 parameters	Δρ_max_ = 0.14 e Å^−^^3^
0 restraints	Δρ_min_ = −0.16 e Å^−^^3^

### Special details

**Table:**

Geometry. All esds (except the esd in the dihedral angle between two l.s. planes) are estimated using the full covariance matrix. The cell esds are taken into account individually in the estimation of esds in distances, angles and torsion angles; correlations between esds in cell parameters are only used when they are defined by crystal symmetry. An approximate (isotropic) treatment of cell esds is used for estimating esds involving l.s. planes.
Refinement. Refinement of F^2^ against ALL reflections. The weighted R-factor wR and goodness of fit S are based on F^2^, conventional R-factors R are based on F, with F set to zero for negative F^2^. The threshold expression of F^2^ > 2sigma(F^2^) is used only for calculating R-factors(gt) etc. and is not relevant to the choice of reflections for refinement. R-factors based on F^2^ are statistically about twice as large as those based on F, and R- factors based on ALL data will be even larger.

### Fractional atomic coordinates and isotropic or equivalent isotropic displacement parameters (Å^2^)

**Table:**

	*x*	*y*	*z*	*U*_iso_*/*U*_eq_	
O1	0.2197 (2)	0.47488 (5)	0.37631 (10)	0.0750 (3)	
O2	0.5718 (2)	0.24790 (4)	0.49538 (12)	0.0737 (3)	
N1	0.17264 (19)	0.52715 (4)	0.19212 (9)	0.0463 (2)	
N2	0.2345 (2)	0.54066 (5)	0.06808 (11)	0.0581 (3)	
N3	0.1096 (2)	0.58305 (5)	0.02364 (12)	0.0640 (3)	
C1	−0.0418 (2)	0.59893 (5)	0.11725 (13)	0.0517 (3)	
C2	−0.2135 (3)	0.64152 (6)	0.11304 (15)	0.0647 (4)	
H2	−0.2396	0.6657	0.0410	0.078*	
C3	−0.3418 (3)	0.64602 (7)	0.21992 (16)	0.0680 (4)	
H3	−0.4572	0.6741	0.2207	0.082*	
C4	−0.3036 (3)	0.60952 (6)	0.32785 (15)	0.0639 (4)	
H4	−0.3955	0.6139	0.3982	0.077*	
C5	−0.1351 (3)	0.56739 (6)	0.33368 (13)	0.0538 (3)	
H5	−0.1104	0.5431	0.4056	0.065*	
C6	−0.0036 (2)	0.56326 (5)	0.22524 (11)	0.0445 (3)	
C7	0.2792 (2)	0.48237 (5)	0.26753 (12)	0.0482 (3)	
C8	0.4576 (2)	0.44711 (5)	0.20553 (13)	0.0522 (3)	
H8A	0.6154	0.4661	0.2081	0.063*	
H8B	0.3910	0.4401	0.1122	0.063*	
C9	0.5001 (2)	0.39358 (5)	0.28009 (12)	0.0463 (3)	
C10	0.7140 (2)	0.38340 (6)	0.36909 (14)	0.0553 (3)	
H10	0.8398	0.4096	0.3802	0.066*	
C11	0.7477 (2)	0.33526 (6)	0.44275 (14)	0.0559 (3)	
H11	0.8941	0.3294	0.5020	0.067*	
C12	0.5628 (2)	0.29645 (5)	0.42717 (13)	0.0501 (3)	
C13	0.3460 (2)	0.30573 (6)	0.33780 (14)	0.0548 (3)	
H13	0.2207	0.2795	0.3265	0.066*	
C14	0.3161 (2)	0.35371 (5)	0.26571 (13)	0.0515 (3)	
H14	0.1698	0.3595	0.2063	0.062*	
C15	0.7849 (3)	0.23650 (8)	0.59061 (19)	0.0839 (5)	
H15A	0.9308	0.2370	0.5469	0.126*	
H15B	0.7676	0.2011	0.6293	0.126*	
H15C	0.8010	0.2637	0.6602	0.126*	

### Atomic displacement parameters (Å^2^)

**Table:**

	*U*^11^	*U*^22^	*U*^33^	*U*^12^	*U*^13^	*U*^23^
O1	0.1114 (9)	0.0677 (7)	0.0547 (6)	0.0257 (6)	0.0415 (6)	0.0192 (5)
O2	0.0810 (7)	0.0529 (6)	0.0814 (7)	−0.0002 (5)	−0.0071 (6)	0.0172 (5)
N1	0.0552 (5)	0.0463 (5)	0.0402 (5)	−0.0011 (4)	0.0166 (4)	0.0041 (4)
N2	0.0705 (7)	0.0606 (7)	0.0485 (6)	0.0056 (5)	0.0264 (5)	0.0127 (5)
N3	0.0781 (8)	0.0648 (7)	0.0534 (6)	0.0118 (6)	0.0244 (6)	0.0174 (5)
C1	0.0591 (7)	0.0498 (7)	0.0473 (6)	−0.0008 (5)	0.0118 (5)	0.0039 (5)
C2	0.0755 (9)	0.0577 (8)	0.0610 (8)	0.0103 (7)	0.0102 (7)	0.0080 (7)
C3	0.0720 (9)	0.0591 (9)	0.0735 (10)	0.0127 (7)	0.0128 (7)	−0.0067 (7)
C4	0.0719 (9)	0.0647 (9)	0.0591 (8)	0.0034 (7)	0.0230 (7)	−0.0109 (7)
C5	0.0671 (8)	0.0536 (7)	0.0432 (6)	−0.0022 (6)	0.0168 (6)	−0.0032 (5)
C6	0.0509 (6)	0.0425 (6)	0.0409 (6)	−0.0059 (5)	0.0092 (5)	−0.0029 (5)
C7	0.0583 (7)	0.0450 (6)	0.0438 (6)	−0.0031 (5)	0.0160 (5)	0.0044 (5)
C8	0.0561 (7)	0.0517 (7)	0.0525 (7)	−0.0008 (5)	0.0203 (5)	0.0058 (5)
C9	0.0460 (6)	0.0478 (6)	0.0475 (6)	0.0016 (5)	0.0149 (5)	0.0007 (5)
C10	0.0427 (6)	0.0570 (8)	0.0664 (8)	−0.0075 (5)	0.0089 (5)	0.0013 (6)
C11	0.0431 (6)	0.0616 (8)	0.0608 (8)	0.0038 (5)	0.0007 (5)	0.0016 (6)
C12	0.0540 (6)	0.0449 (6)	0.0513 (7)	0.0041 (5)	0.0075 (5)	−0.0001 (5)
C13	0.0527 (7)	0.0493 (7)	0.0603 (7)	−0.0088 (5)	0.0008 (6)	0.0007 (6)
C14	0.0473 (6)	0.0539 (7)	0.0514 (6)	−0.0020 (5)	0.0011 (5)	0.0014 (5)
C15	0.0787 (10)	0.0842 (12)	0.0863 (11)	0.0217 (9)	0.0046 (9)	0.0308 (10)

### Geometric parameters (Å, º)

**Table:**

O1—C7	1.1997 (14)	C7—C8	1.4997 (18)
O2—C12	1.3700 (16)	C8—C9	1.5106 (17)
O2—C15	1.414 (2)	C8—H8A	0.9700
N1—C6	1.3785 (16)	C8—H8B	0.9700
N1—N2	1.3792 (13)	C9—C10	1.3783 (18)
N1—C7	1.4075 (16)	C9—C14	1.3879 (17)
N2—N3	1.2829 (16)	C10—C11	1.3898 (19)
N3—C1	1.3928 (17)	C10—H10	0.9300
C1—C6	1.3849 (17)	C11—C12	1.3732 (18)
C1—C2	1.394 (2)	C11—H11	0.9300
C2—C3	1.368 (2)	C12—C13	1.3878 (18)
C2—H2	0.9300	C13—C14	1.3775 (18)
C3—C4	1.398 (2)	C13—H13	0.9300
C3—H3	0.9300	C14—H14	0.9300
C4—C5	1.373 (2)	C15—H15A	0.9600
C4—H4	0.9300	C15—H15B	0.9600
C5—C6	1.3924 (17)	C15—H15C	0.9600
C5—H5	0.9300		
			
C12—O2—C15	118.34 (13)	C9—C8—H8A	109.5
C6—N1—N2	109.58 (10)	C7—C8—H8B	109.5
C6—N1—C7	127.83 (10)	C9—C8—H8B	109.5
N2—N1—C7	122.59 (10)	H8A—C8—H8B	108.1
N3—N2—N1	108.80 (10)	C10—C9—C14	117.56 (12)
N2—N3—C1	108.91 (10)	C10—C9—C8	122.04 (11)
C6—C1—N3	108.63 (11)	C14—C9—C8	120.32 (11)
C6—C1—C2	121.12 (12)	C9—C10—C11	122.05 (12)
N3—C1—C2	130.24 (12)	C9—C10—H10	119.0
C3—C2—C1	116.83 (13)	C11—C10—H10	119.0
C3—C2—H2	121.6	C12—C11—C10	119.37 (12)
C1—C2—H2	121.6	C12—C11—H11	120.3
C2—C3—C4	121.63 (14)	C10—C11—H11	120.3
C2—C3—H3	119.2	O2—C12—C11	124.94 (12)
C4—C3—H3	119.2	O2—C12—C13	115.46 (12)
C5—C4—C3	122.31 (13)	C11—C12—C13	119.60 (12)
C5—C4—H4	118.8	C14—C13—C12	120.17 (12)
C3—C4—H4	118.8	C14—C13—H13	119.9
C4—C5—C6	115.84 (13)	C12—C13—H13	119.9
C4—C5—H5	122.1	C13—C14—C9	121.25 (12)
C6—C5—H5	122.1	C13—C14—H14	119.4
N1—C6—C1	104.08 (10)	C9—C14—H14	119.4
N1—C6—C5	133.61 (12)	O2—C15—H15A	109.5
C1—C6—C5	122.28 (12)	O2—C15—H15B	109.5
O1—C7—N1	117.76 (11)	H15A—C15—H15B	109.5
O1—C7—C8	124.65 (12)	O2—C15—H15C	109.5
N1—C7—C8	117.59 (10)	H15A—C15—H15C	109.5
C7—C8—C9	110.69 (10)	H15B—C15—H15C	109.5
C7—C8—H8A	109.5		
			
C6—N1—N2—N3	0.65 (15)	C6—N1—C7—O1	−2.9 (2)
C7—N1—N2—N3	−179.84 (12)	N2—N1—C7—O1	177.64 (13)
N1—N2—N3—C1	−0.39 (16)	C6—N1—C7—C8	176.55 (11)
N2—N3—C1—C6	0.00 (16)	N2—N1—C7—C8	−2.87 (17)
N2—N3—C1—C2	−178.46 (15)	O1—C7—C8—C9	14.73 (19)
C6—C1—C2—C3	−0.3 (2)	N1—C7—C8—C9	−164.72 (11)
N3—C1—C2—C3	178.03 (15)	C7—C8—C9—C10	−103.08 (14)
C1—C2—C3—C4	−0.3 (2)	C7—C8—C9—C14	73.62 (15)
C2—C3—C4—C5	0.4 (3)	C14—C9—C10—C11	−0.1 (2)
C3—C4—C5—C6	0.1 (2)	C8—C9—C10—C11	176.70 (12)
N2—N1—C6—C1	−0.62 (13)	C9—C10—C11—C12	−0.1 (2)
C7—N1—C6—C1	179.91 (12)	C15—O2—C12—C11	0.8 (2)
N2—N1—C6—C5	177.24 (13)	C15—O2—C12—C13	−178.41 (14)
C7—N1—C6—C5	−2.2 (2)	C10—C11—C12—O2	−178.87 (13)
N3—C1—C6—N1	0.38 (14)	C10—C11—C12—C13	0.3 (2)
C2—C1—C6—N1	179.01 (13)	O2—C12—C13—C14	178.92 (12)
N3—C1—C6—C5	−177.78 (12)	C11—C12—C13—C14	−0.3 (2)
C2—C1—C6—C5	0.8 (2)	C12—C13—C14—C9	0.1 (2)
C4—C5—C6—N1	−178.29 (13)	C10—C9—C14—C13	0.06 (19)
C4—C5—C6—C1	−0.75 (19)	C8—C9—C14—C13	−176.78 (12)

### Hydrogen-bond geometry (Å, º)

**Table:**

*D*—H···*A*	*D*—H	H···*A*	*D*···*A*	*D*—H···*A*
C5—H5···O1^i^	0.93	2.40	3.1912 (16)	143

Symmetry code: (i) −*x*, −*y*+1, −*z*+1.
